# Supplementary material for: A genome catalog of the early-life human skin microbiome
Source: Genome Biol. 2023 Nov 10;24:252. doi: 10.1186/s13059-023-03090-w (PMC10636849; doi:10.1186/s13059-023-03090-w)
Supplement: Supplementary file 2 — Additional file 2: Figure S1. Quality metrics of the nonredundant genomes in the ELSG catalog. Figure S2. Expansion of species diversity in the ELSG catalog. Figure S3. Early-life skin microbial community structure. Figure S4. Proteins and functions of early-life skin microbiome. Figure S5. Intraspecies single-nucleotide variation and mother-infant strain sharing. [file 13059_2023_3090_MOESM2_ESM.pdf]

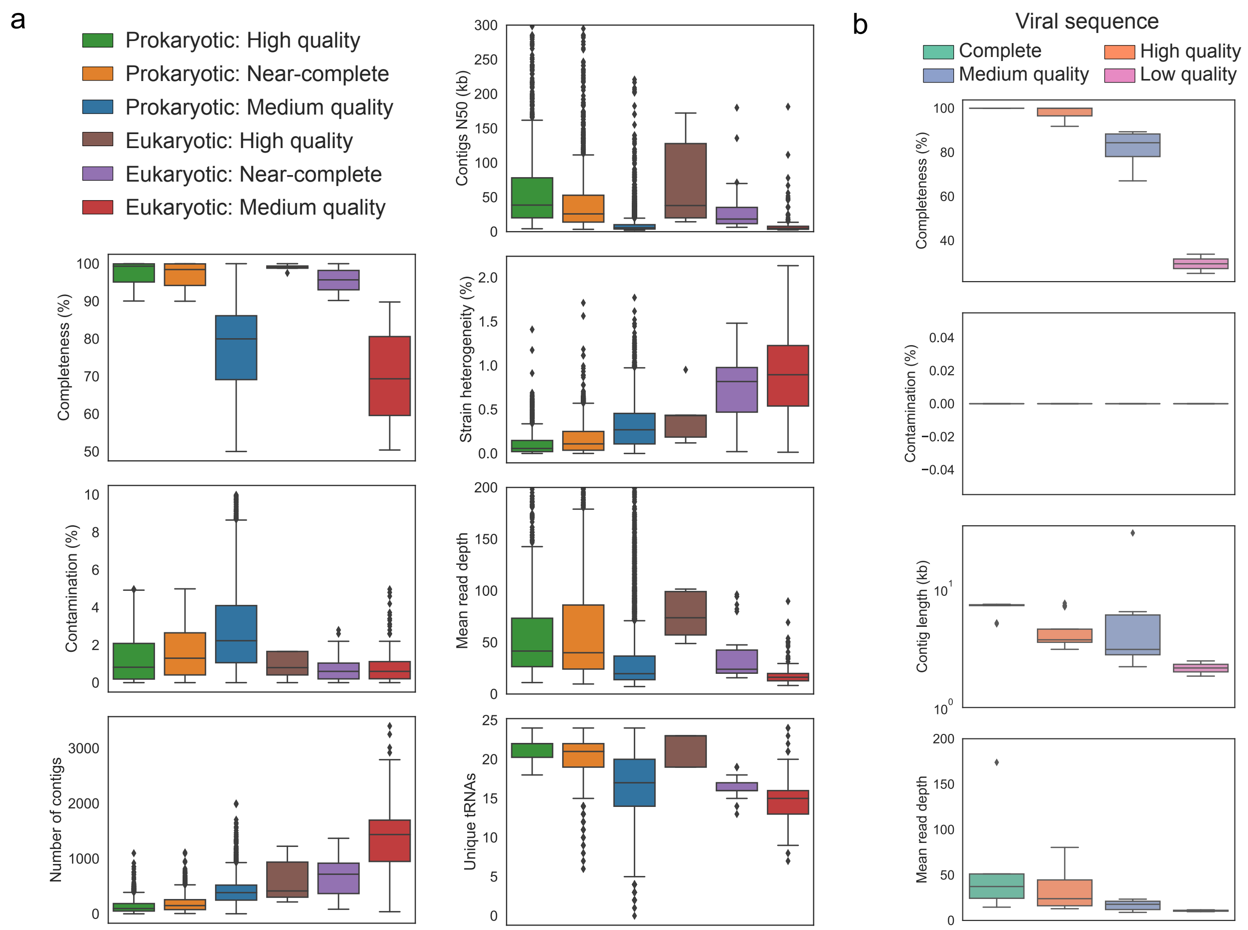

Figure S1. Quality metrics of the nonredundant genomes in the ELSG catalog.

a. Genomes were stratified by quality level with colors matching those in Figure 1. Box lengths represent the interquartile range, and whiskers indicate the lowest and highest values within 1.5 times the interquartile range.

b. Eukaryotic viral sequences were stratified by quality level with colors matching those in Figure 1.

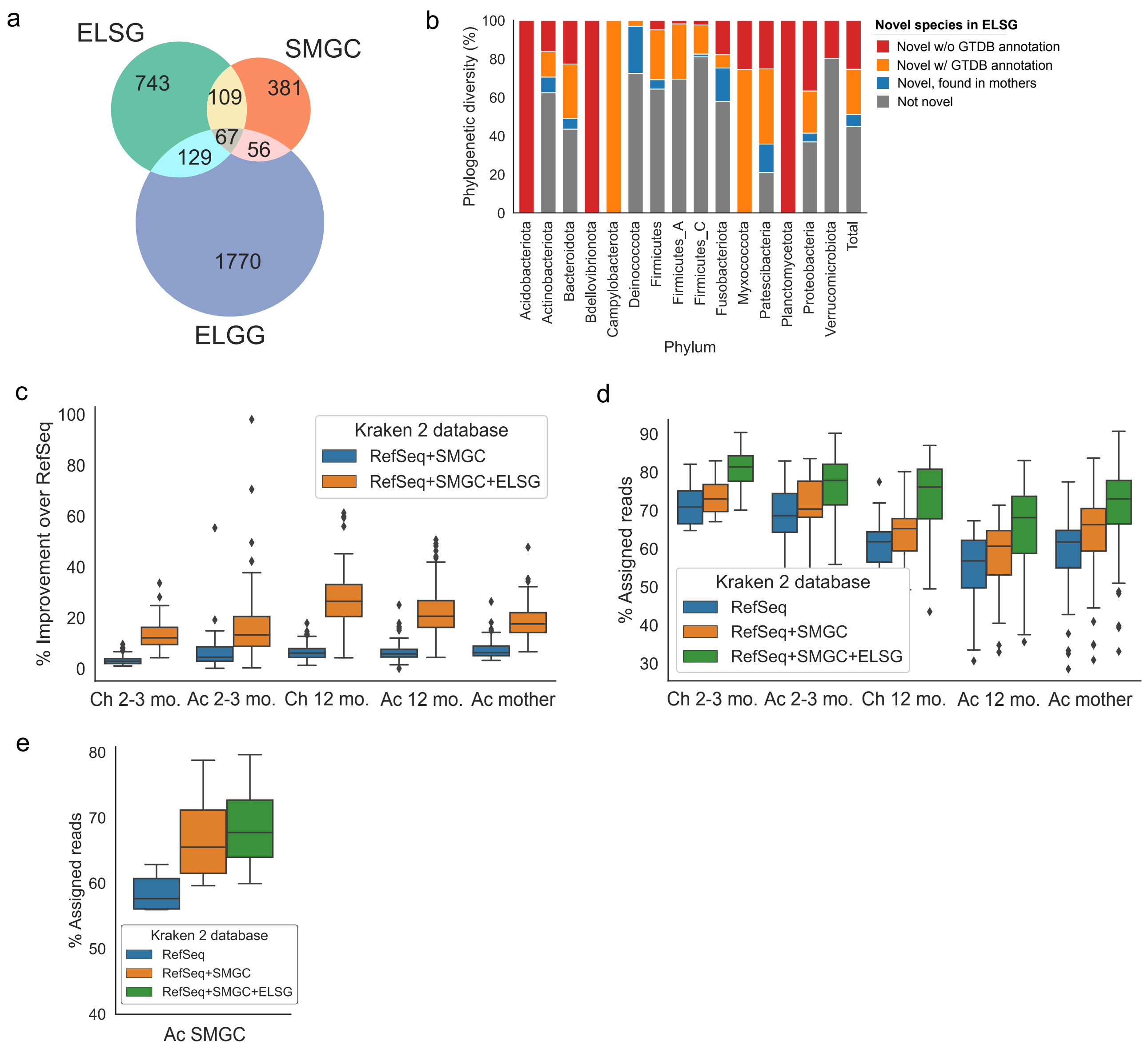

Figure S2. Expansion of species diversity in the ELSG catalog.

a. Comparison of species-level representative MAGs from three genome catalogs: ELSG, SMGC, and ELGG. The numbers indicate MAGs from each catalog that did or did not cluster with other catalogs at the species level.
b. Phylogenetic diversity accounted by known and novel species. Colors match Figure 2.
c. The improvement rate of read classification over the standard Kraken 2 RefSeq database.
d. Kraken 2 read classification rate of skin samples that did not directly contribute MAGs to the ELSG.
e. Kraken 2 classification rate of reads from published skin metagenomic data.

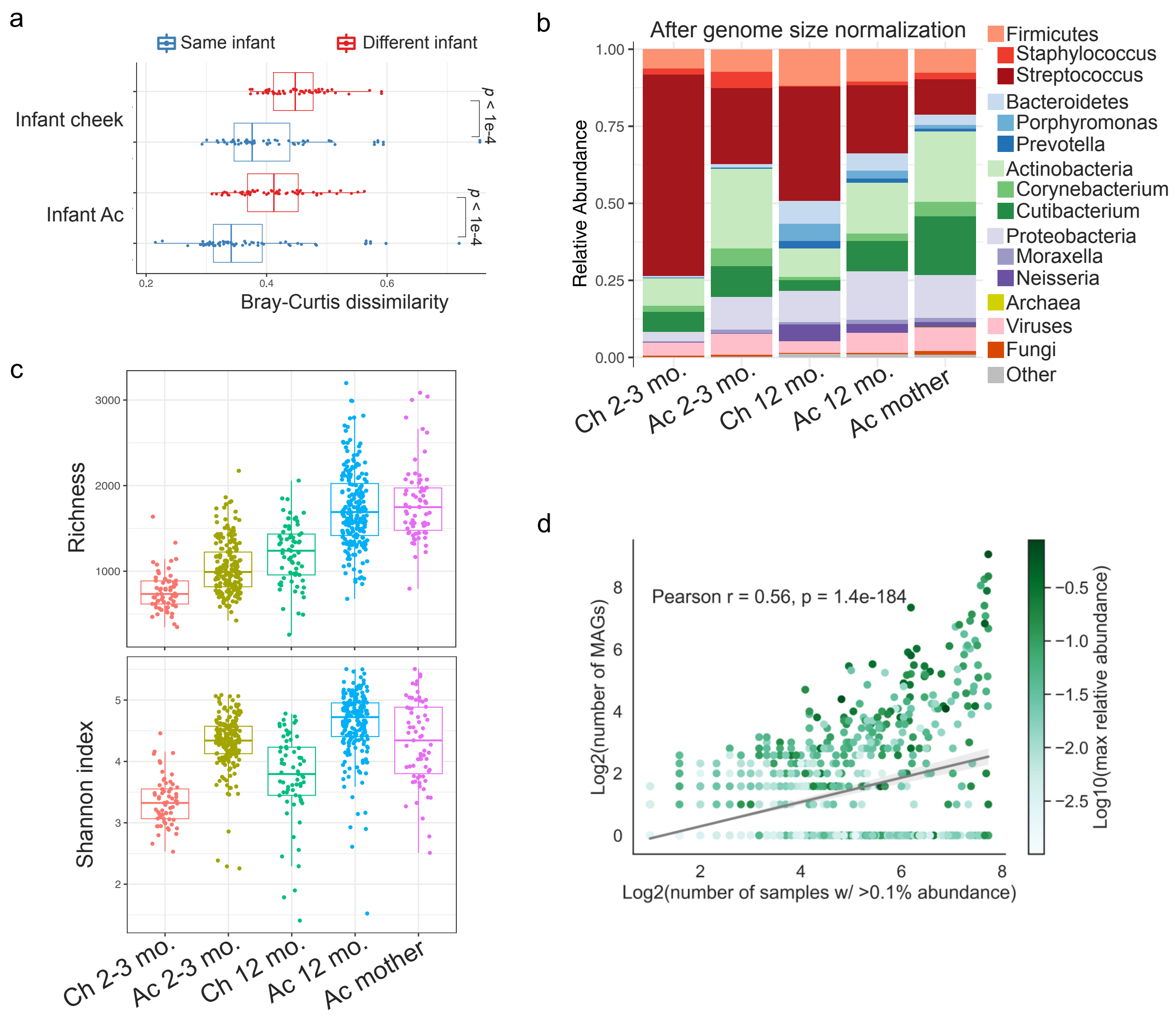

Figure S3. Early-life skin microbial community structure.

a. The Bray-Curtis dissimilarity of microbial community between two time points of the same infant compared with that of different infants. Median value of each infant and all other infants was used to plot. Statistical difference was tested by two-sided Wilcoxon rank sum test.
b. Relative abundance of skin microbiome averaged for each sample group after genome size normalization, which emphasized the viral community.
c. Alpha diversity (richness and Shannon index) of skin samples divided by age group and skin site.
d. Relationship between species prevalence and number of MAGs. Each dot represents a species, the color of which indicates maximum relative abundance among all samples. The prevalence of a species was calculated as the number of samples with >0.1% relative abundance of that species. Pearson's correlation coefficient and p-value indicate a significant correlation.

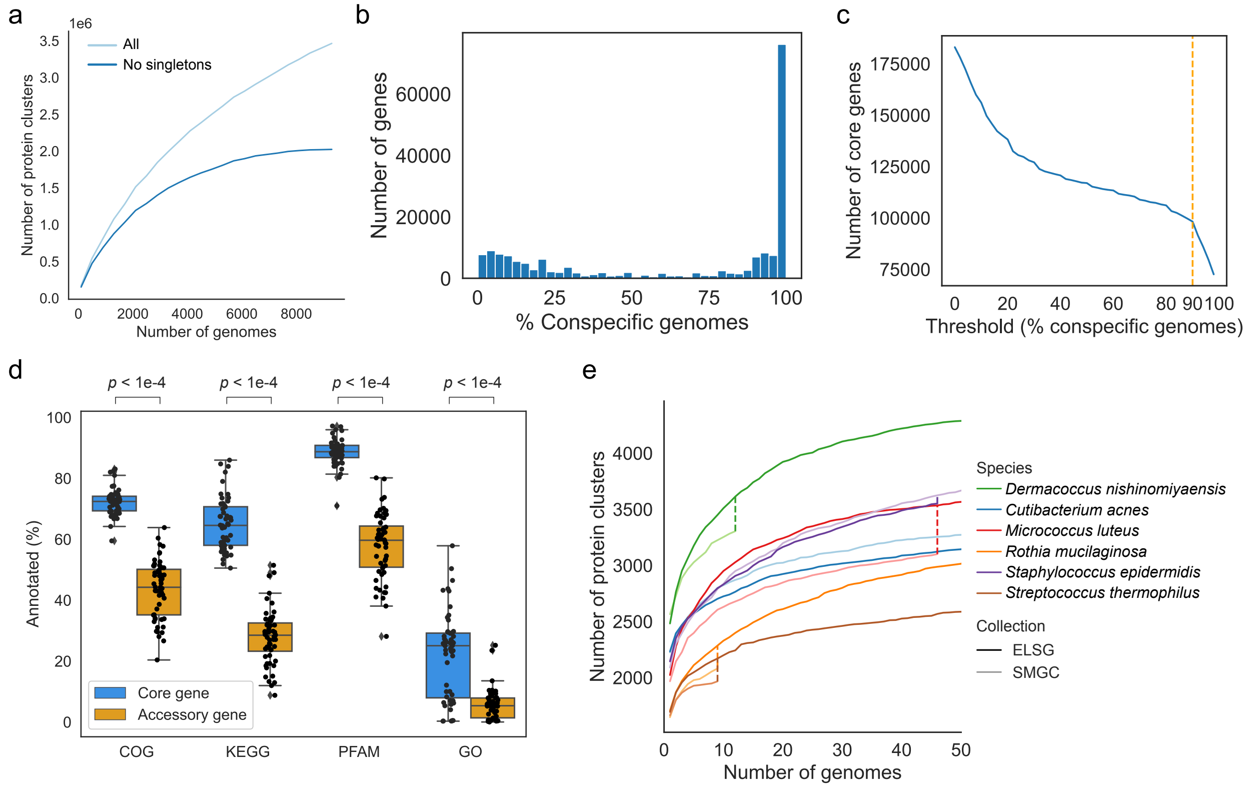

Figure S4. Proteins and functions of early-life skin microbiome.

a. Rarefaction curves of the number of protein clusters as a function of the number of genomes for all species combined. Separate curves are depicted for proteins clustered at 90% amino acid identity for all protein clusters and after excluding singleton protein clusters (containing only one protein sequence).
b. The number of genes in relation to the fraction of conspecific genomes where genes were found. Only near-complete and high-quality genomes were considered in the analysis.
c. Number of genes shared by conspecific genomes in relation to the cutoff on the fraction of conspecific genomes. Vertical dashed line represents the 90% threshold used in this study to define core genes.
d. Proportion of core and accessory genes annotated by various databases including Clusters of Orthologous Genes (COG), Kyoto Encyclopedia of Genes and Genomes (KEGG), Pfam, and Gene Ontology (GO). Each dot represents a species. Only species with at least 10 near-complete or high-quality genomes are included. Statistical significance was tested by two-tailed Wilcoxon rank sum test.
e. Comparison of the rate of gene gain between the ELSG and the SMGC. Only near-complete and high-quality genomes are included. Dashed line connects the end point of the collection with fewer number of genomes of the same species.

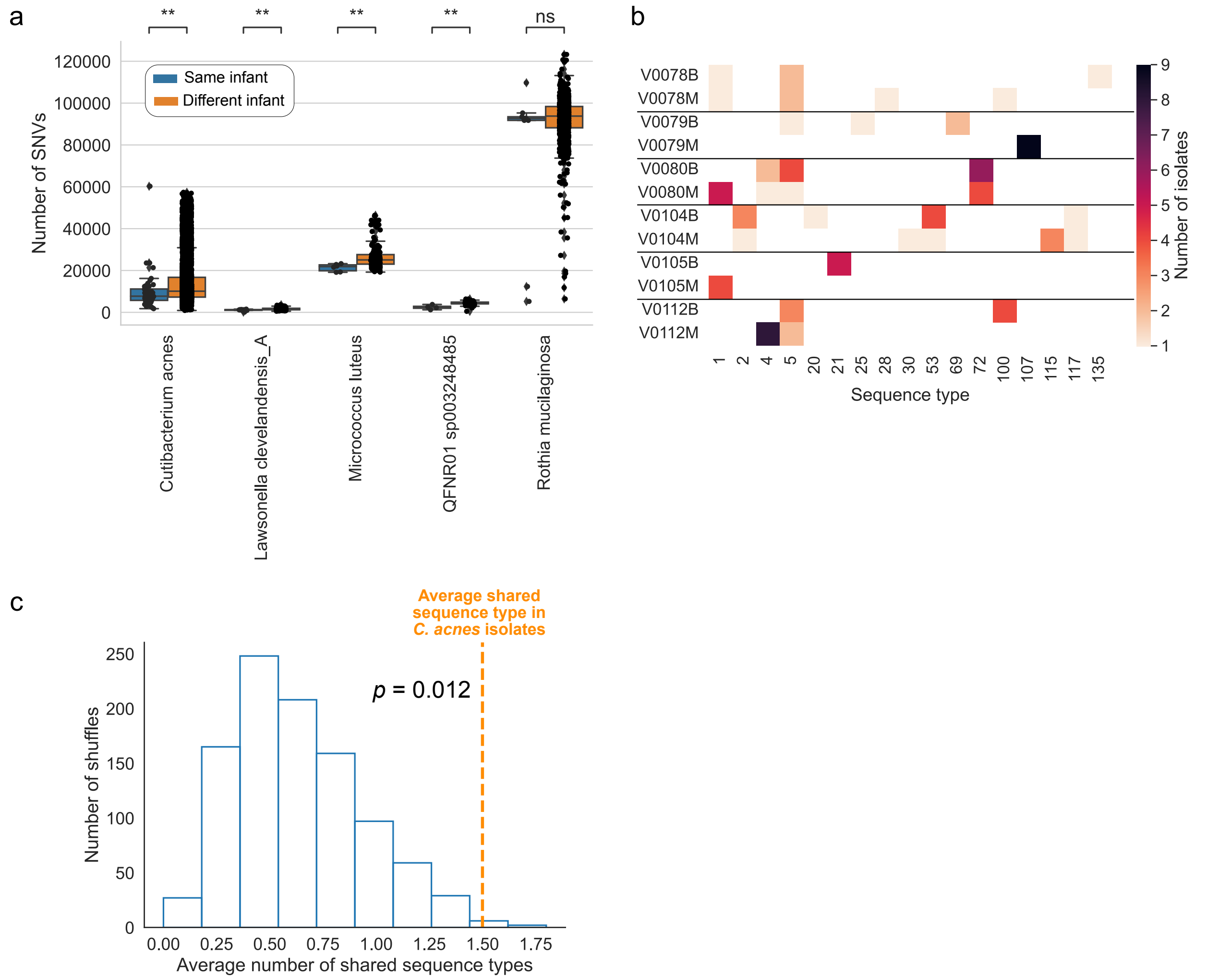

Figure S5. Intraspecies single-nucleotide variation and mother-infant strain sharing.

a. Number of SNVs in pairwise comparisons between genomes of the same infant at 2-3 months and 12 months (same infant) and between any two genomes assembled from different times (different infant). Only species with genomes from at least 3 infants at different times were considered for analysis. Statistical significance was tested by two-tailed Wilcoxon rank sum test. *P<0.05, **P<0.01. Non-significance ("ns") indicates P>0.05.
b. Number of sequence types of *C. acnes* cultured isolates from each individual.
c. Average number of shared sequence types of *C. acnes* cultured isolates between related infants and mothers (orange dashed line) and between any two individuals after 1,000 permutations (histogram).
