# Supplementary material for: A genome catalog of the early-life human skin microbiome
Source: Genome Biol. 2023 Nov 10;24:252. doi: 10.1186/s13059-023-03090-w (PMC10636849; doi:10.1186/s13059-023-03090-w)
Supplement: Supplementary file 3 — Additional file 3. Review history. [file 13059_2023_3090_MOESM3_ESM.docx]

Review History

**First round of review**

**Reviewer 1**

**Were you able to assess all statistics in the manuscript, including the appropriateness of statistical tests used?**

Yes, and I have assessed the statistics in my report.

**Comments to author:**

In this study, the authors present an Early-Life Skin Genomes (ELSG) catalog based on skin metagenome sequencing data from Australian infants. This catalog contains bacterial, fungal, and eukaryotic viral sequences, revealing many novel species not previously known to part of the human skin microbiome. The authors provide taxonomic profiles of the skin microbiome of Australian infants, highlighting the characteristics of the infant skin microbiome. Additionally, the authors offer not only the genome catalog but also the protein catalog, presenting functional features of the infant skin microbiome. The study also provides evidence for vertical transmission of the skin microbiome between mother and infant through SNV analysis.

As stated by the authors, the ELSG catalog represents the first infant skin microbiome genome database. An important finding is the evidence of previously underexplored mother-to-infant transmission of the skin microbiome, as supported by the analysis involving infant-matched mothers. However, a major concern is that the catalog is based solely on Australian infant samples, raising questions about the generalizability of the observations to the human infant skin microbiome as a whole. Therefore, before this paper can be accepted for publication. the following questions need to be addressed.

[Major comments]

1. Early life gut genomes (ELGG) catalog contains MAGs derived from infant fecal samples in 26 studies spanning multiple countries. In contrast, the ELSG is based exclusively on Australian infant samples. It is important to determine to which extent the findings of this study can be generalized for human infant skin microbiome, given the limited geographic scope of the data.

2. The authors found a eukaryotic virus by aligning contigs to nt database with BLASTn. There are many machine learning and deep learning methods for detecting viral contigs, some of which can also identify phage genomes as well. Is there any reason for focusing exclusively on eukaryotic viruses and not including prokaryotic viruses in the analysis?

3. The authors use 'lineage_wf' workflow of CheckM v1.1.3 to check the quality of refined bins. Although CheckM has been a popular tool for bin quality control, 'lineage_wf' workflow in CheckM v1 results in low accuracy and sensitivity for novel lineages with less robust marker sets. To address this issue, there is an upgraded tool, CheckM v2. Have you tried CheckM v2? In Figure 2f:

4. The authors show the improvement of the read classification rate using metagenome sequencing samples employed in the construction of ELSG. However, to validate this improvement, it would be necessary to use metagenome sequencing samples not used in the construction of ELSG.

5. Line 172, "Together these findings suggest fungal specificity of early-life skin". There is a difference in age between SMGC and ELSG, but there is also a difference in population. Consequently, it is challenging to claim fungal specificity in early-life skin with certainty. To support this claim, a comparison of fungi obtained from infant-matched mothers should also be included.

6. Line 281-283, "To further validate the mother-infant transmission of skin microbiome, we cultured Cutibacterium acnes from the nasal swabs collected from six pairs of infants and mothers when infants were 12-month-old". How can this claim be justified as a validation of mother-infant transmission of the skin microbiome if the samples collected were nasal swabs?

7. Line 500. Differential abundance analysis by DEseq2 is not widely used in microbiome research. Given that microbiome data is compositionally constrained and compositionality can significantly impact the result of differential abundance of species, it is recommended that the authors use alternative methods that consider compositionality, such as ANCOM-BC?

[Minor comments]

1. Line 152, "699 clustered independently of any genome from the SMGC and the ELGG". However, the Venn diagram in Fig. S2a shows that this part is 690. Please clarify the correct value. Additionally, the sum of the numbers in the ELSG Venn diagram does not equal 1,029.

2. Line 165-166, "Malassezia was unsurprisingly the dominant genus". This sentence appears to be based on the fact that Malassezia is the most prevalent fungus identified in the human skin microbiome. Please provide a brief explanation of this and include a reference to support the claim.

3. Line 277, "~across individuals (Fig. S5)". Replace 'Fig. S5' by 'Fig. S5a'.

4. Line 449-450. "fastANI v1.33 was used to accelerate the process". The authors set the '--SkipSecondary' parameter, but it is unclear whether fastANI, the algorithm for secondary clustering comparisons, can actually accelerate this process. It is recommended to either remove this sentence or include it in the paragraph below that discusses the actual secondary clustering.

5. Line 541-542. "The raw metagenomic sequencing data are available in the NCBI BioProject database under project number PRJNA971252". The corresponding BioProject number does not exist.

6. Line 546-547. "All the code utilized in this study is available on GitHub at https://github.com/skinmicrobiome/ELSG". The webpage is unavailable.

7. Figure S1b: According to the manuscript, eukaryotic viral sequences were >1000bp (line 442). However, in the third boxplot of Figure S1(b), y-axis said the contig length is lower than 10-2 kb. Please address this discrepancy.

8. Figure 2: The figure has no scale of the bar graphs in the outermost layer.

9. In line 277, Fig. S5 has to be changed to Fig. S5a

10. 441-443 "Eukaryotic viral sequences were detected by aligning the contigs from MEGAHIT to the nt database with BLASTn v2.8.032 by requiring >90% nucleotide identity, >1000 bp aligned sequence, and >70% total contig aligned". This sentence is redundant with the information provided in lines 432-434.

11. Line 522. What was minimum number of conspecific genomes for SNV analysis?

**Reviewer 2**

**Were you able to assess all statistics in the manuscript, including the appropriateness of statistical tests used?**

Yes, and I have assessed the statistics in my report.

**Comments to author:**

In this study, Shen and colleague collected skin samples from infants at age 2-3 months and 12 months for ultra-deep shotgun metagenomic sequencing to generate the early-life skin microbial genome catalog. Afterwards, based on the built catalogue, the author analyzed the property of the early-life skin microbiome. The comparisons between infants and mothers were conducted. Overall, this method is valid and the results of work are quite interesting and important for the future research from the early life.

Major comments:

1. From the title, the author aimed to build a catalogue of skin microbiome for infants. However, the infants are only from Australia, and the included subjects are not too many. As there have are other skin metagenomes from infants, I would suggest the author to search and include these public skin datasets from infants and incorporate them with the datasets newly sequenced in this study. Also, the rarefaction analysis showed that the number of species in the ELSG was not saturated, although the situation is better after removing the rare species.

2. The mother-to-infant transmission have been studied for the gut microbiome, as the author mentioned as well. Different from the gut microbiome that mainly comprises the strict anaerobic microbes, which means that these microbes are hard to survive in the environment, the skin microbiome are not anaerobic, and also fully exposed to the environment. Although the same species/strain shared by mothers and infants, how can the author exclude the possibility that these microbes are not transferred from environments to mothers and infants as they are shared the same environments. Therefore, the author should address in the discussion or be careful to use "transmission".

3. In "Comparison of taxonomic profiles between early-life and adult skin microbiome" part, the author should also describe and discuss the profile of microbiome from mother skin, and the differential taxa associated with mothers and infants. Only beta diversity comparison can provide quite limited information.

4. For microbial annotation, the author used the RefSeq database (release 99), which was released three years ago. I understand that that RefSeq is frequently updated, but I do not why the author used this very old release version, although I am not sure when the author conducted the analysis. The scientific reason has to be mentioned. However, I strongly recommend the author to use the updated release.

Minor comments:

Line 28: how does the "ultra-deep" shotgun sequences defined?

Line 263: only 7 species were shared by mother-infant?

Line 266: what the author means with "consistent with vertical transmission of skin microbes between mother and infant"? is it only based on the lower SNVs of paired mother-infants than unrelated ones? Is there a cut-off to define the transmission? Again, the "transmission" should be used with caution.

Line 183: please update the release of RefSeq and see whether any difference rather than the 77% improvement.

Line 491-495: is there any reason to exclude samples less than 800,000 classified reads and also 20% for meta diversity?

Line 411: is there any viral sequences that are removed after assessing by CheckV?

Line 546: the link for code availability is not valid. Please update.

**Authors Response**

**Point-by-point responses to the reviewers’ comments:**

Reviewer #1:

In this study, the authors present an Early-Life Skin Genomes (ELSG) catalog based on skin metagenome sequencing data from Australian infants. This catalog contains bacterial, fungal, and eukaryotic viral sequences, revealing many novel species not previously known to part of the human skin microbiome. The authors provide taxonomic profiles of the skin microbiome of Australian infants, highlighting the characteristics of the infant skin microbiome. Additionally, the authors offer not only the genome catalog but also the protein catalog, presenting functional features of the infant skin microbiome. The study also provides evidence for vertical transmission of the skin microbiome between mother and infant through SNV analysis.

As stated by the authors, the ELSG catalog represents the first infant skin microbiome genome database. An important finding is the evidence of previously underexplored mother-to-infant transmission of the skin microbiome, as supported by the analysis involving infant-matched mothers. However, a major concern is that the catalog is based solely on Australian infant samples, raising questions about the generalizability of the observations to the human infant skin microbiome as a whole. Therefore, before this paper can be accepted for publication. the following questions need to be addressed.

[Major comments]

1. Early life gut genomes (ELGG) catalog contains MAGs derived from infant fecal samples in 26 studies spanning multiple countries. In contrast, the ELSG is based exclusively on Australian infant samples. It is important to determine to which extent the findings of this study can be generalized for human infant skin microbiome, given the limited geographic scope of the data.

*Reply: We appreciate the valuable insights provided by Reviewer #1. As highlighted, there is indeed a notable body of research encompassing infant gut metagenomes from multiple countries. However, when considering the realm of infant skin metagenomes, it is important to clarify that the prevailing majority of published studies have predominantly employed amplicon sequencing techniques, despite the occasional use of the term "metagenomics" (e.g., BioProject PRJNA833324), which may lead to confusion with the shotgun metagenomic sequencing approach undertaken in our study. It is noteworthy to mention that the inclusion of shotgun metagenomic data pertaining to infant skin is relatively scarce in the literature. For the very few studies with shotgun metagenomic data, some contend with small sample sizes (e.g., n=2 within BioProject PRJNA573942, which primarily focuses on the gut microbiome, or n=10 from BioProject PRJNA937622 serving the purpose of protocol validation). Others focused on NICUs (neonatal intensive care units), as observed in PMID: 37422700, where the data deposition is still pending. Given the restricted availability of relevant studies concerning full-term infants' skin metagenomes—such as those studied in our research—we opted to establish the ELSG catalog exclusively utilizing our collected samples. Acknowledging the limitation stemming from the exclusive focus on Australian infants in our sample collection, we have addressed this concern transparently within the Discussion section of the revised manuscript.*

2. The authors found a eukaryotic virus by aligning contigs to nt database with BLASTn. There are many machine learning and deep learning methods for detecting viral contigs, some of which can also identify phage genomes as well. Is there any reason for focusing exclusively on eukaryotic viruses and not including prokaryotic viruses in the analysis?

*Reply: We acknowledge reviewer’s suggestion of identifying viral contigs including phages using machine learning-based tools (e.g., VirFinder, VirSorter, etc.). However, we decided to not overcrowd the ELSG catalog with potentially thousands of viral contigs, which would have been predicted by these tools but not robustly assigned with taxonomy lower than the family level. Instead, we focused on eukaryotic viruses with confident taxonomic classifications (using BLASTn to align to existing genomes), which could add value to our catalog in the context of infectious diseases that might affect infants. We acknowledge that the ELSG catalog is missing phages and likely novel eukaryotic viruses, but we were conservative to avoid false positives or ambiguous viral sequences in our catalog. To clarify our intention, we included the rationale of detecting eukaryotic viruses in the revised manuscript: “To elucidate viruses with a higher likelihood of affecting infants and causing infectious diseases”.*

3. The authors use 'lineage_wf' workflow of CheckM v1.1.3 to check the quality of refined bins. Although CheckM has been a popular tool for bin quality control, 'lineage_wf' workflow in CheckM v1 results in low accuracy and sensitivity for novel lineages with less robust marker sets. To address this issue, there is an upgraded tool, CheckM v2. Have you tried CheckM v2? In Figure 2f:

*Reply: CheckM2 was recently published in a peer-reviewed journal after we received these reviews, so we appreciate reviewer’s insightful suggestion. We were impressed by the benchmarking results of CheckM2, so we decided to extend CheckM2 to our MAGs to re-assess the completeness and contamination. We found that CheckM1 and CheckM2 have high correlations for the original ELSG catalog (see below; Pearson r=0.87 for completeness and r=0.72 for contamination).*

*Despite the high correlations, there are MAGs that were predicted to have higher completeness and lower contamination with CheckM2, which could be critical for novel MAGs that would have passed the MIMAG cutoffs based on CheckM2. Thus, we decided to run CheckM2 on all the MAGs and expanded the original ELSG catalog to include an additional 289 non-redundant prokaryotic MAGs with >50% completeness and <10% contamination based on CheckM2, now reaching 9,483 prokaryotic MAGs. Supplemental table 3 now includes both CheckM1 and CheckM2 metrices. By presenting this more inclusive genome catalog, we believe it will serve the community better especially for those who would like more flexibility with doing personalized filtering based on either CheckM1 or CheckM2 statistics or both. The results and texts have been revised accordingly based on the new ELSG catalog.*

4. The authors show the improvement of the read classification rate using metagenome sequencing samples employed in the construction of ELSG. However, to validate this improvement, it would be necessary to use metagenome sequencing samples not used in the construction of ELSG.

*Reply: As mentioned in our response to your first comment, we are unaware of any other relevant shotgun metagenomic data for infant skin besides this study, which made the ELSG the first genome catalog for infant skin microbiome. To validate the improvement of the read classification rate, we separated out the 200 samples that did not directly contribute any MAGs to the ELSG catalog and recreated another version of Figure 2F for these samples (see below). We saw that despite being independent from the ELSG catalog, these samples still showed a median of 75% read classification rate and 17% improvement. We have included this new result as a new Figure S2d in the revised manuscript. In addition, we added a discussion point stating that “the validation of the ELSG as an effective resource of improving read classification for infants from distinct geographic backgrounds is an avenue for future research.”*

*• New Figure S2d: Kraken 2 read classification rate of skin samples that did not directly contribute MAGs to the ELSG.*

5. Line 172, "Together these findings suggest fungal specificity of early-life skin". There is a difference in age between SMGC and ELSG, but there is also a difference in population. Consequently, it is challenging to claim fungal specificity in early-life skin with certainty. To support this claim, a comparison of fungi obtained from infant-matched mothers should also be included.

*Reply: We appreciate the reviewer's thoughtful consideration of the age and population differences between the SMGC and ELSG groups in our study. As suggested by the reviewer, we assembled fungal MAGs from our mother samples. Malassezia restricta (23 MAGs) and Malassezia globose (2 MAGs) were successfully assembled, both of which were also assembled from infant samples. Several other Malassezia species (e.g., M. arunalokei, M. sympodialis, M. palmae, M. slooffiae) and Saccharomyces were not assembled from mother samples, but we acknowledge that it is challenging to conclude early-life-skin specificity with certainty based on only one sampling site (antecubital fossa) of mother skin. Thus, we have modified the language in the revised manuscript to be “these findings demonstrated the fungal diversity of early-life skin, as well as the commonalities and potential differences between early-life skin and adult skin”.*

6. Line 281-283, "To further validate the mother-infant transmission of skin microbiome, we cultured Cutibacterium acnes from the nasal swabs collected from six pairs of infants and mothers when infants were 12-month-old". How can this claim be justified as a validation of mother-infant transmission of the skin microbiome if the samples collected were nasal swabs?

*Reply: We appreciate reviewer’s insights of a logical gap in our transition from skin metagenomics to nasal culture. While nasal swabs may not directly represent the skin microbiome, there are certain considerations that make this approach a proxy for studying skin microbiome transmission:*

*1) There is frequent microbiome exchange between the nares and the skin, especially in infants. Cutibacterium acnes is commonly found on the skin, so detecting it in nasal swabs suggests a potential transfer of microorganisms from the skin.*

*2) Direct culturing from skin swabs can be challenging. Nasal swabs contain a larger microbial biomass and provide a practical way to indirectly assess microbiome sharing.*

*We have incorporated a transitional statement with the key information above into the revised manuscript to elucidate our rationale for using nasal swabs as a proxy for studying skin microbiome strain sharing.*

7. Line 500. Differential abundance analysis by DEseq2 is not widely used in microbiome research. Given that microbiome data is compositionally constrained and compositionality can significantly impact the result of differential abundance of species, it is recommended that the authors use alternative methods that consider compositionality, such as ANCOM-BC?

*Reply: We appreciate the reviewer's insight into the challenges posed by compositionality in microbiome data analysis. We acknowledge that DESeq2 may not be ideal for analyzing compositional data due to its assumptions even though it was previously recommended by a method review article (PMID: 32746888). In light of this, we ran ANCOM-BC as a parallel analysis on the same dataset as we used for DESeq2. We found that the results generated by the two tools are highly correlated for the top differential taxa displayed in Figure 3d (see below). Despite being interesting, the comparison of different methods is out of the scope of this study, so we kept the original figure unchanged.*

[Minor comments]

1. Line 152, "699 clustered independently of any genome from the SMGC and the ELGG". However, the Venn diagram in Fig. S2a shows that this part is 690. Please clarify the correct value. Additionally, the sum of the numbers in the ELSG Venn diagram does not equal 1,029.

*Reply: We have replaced the original Fig. S2a with the new ELSG catalog by including additional MAGs passing the MIMAG cutoffs based on CheckM2 statistics. In the revised manuscript, we made sure that the number of ELSG-specific clusters stated in texts matches that shown in the Venn diagram. However, the number of clusters with at least one ELSG MAG (1048) still does not equal the original number of ELSG clusters (1055), because by combining three catalogs (ELSG, SMGC, and ELGG), the original clusters based on ELSG alone could be merged due to the additional genomes from the SMGC and/or ELGG, which fell in between two ELSG clusters in terms of sequence similarity as illustrated by the graph below. Therefore, the number of clusters with ELSG MAGs reduces slightly after combining with SMGC and ELGG.*

2. Line 165-166, "Malassezia was unsurprisingly the dominant genus". This sentence appears to be based on the fact that Malassezia is the most prevalent fungus identified in the human skin microbiome. Please provide a brief explanation of this and include a reference to support the claim.

*Reply: We have added a brief explanation and included references as the reviewer suggested: “Malassezia was the predominant genus, consistent with previous studies that found Malassezia as the major fungal genus across multiple skin sites”.*

3. Line 277, "~across individuals (Fig. S5)". Replace 'Fig. S5' by 'Fig. S5a'.

*Reply: We have replaced “Fig. S5” by “Fig. S5a” in the revised manuscript accordingly.*

4. Line 449-450. "fastANI v1.33 was used to accelerate the process". The authors set the '--SkipSecondary' parameter, but it is unclear whether fastANI, the algorithm for secondary clustering comparisons, can actually accelerate this process. It is recommended to either remove this sentence or include it in the paragraph below that discusses the actual secondary clustering.

*Reply: We appreciate reviewer’s insights and have moved "fastANI v1.33 was used to accelerate the process" to where the actual secondary clustering is discussed.*

5. Line 541-542. "The raw metagenomic sequencing data are available in the NCBI BioProject database under project number PRJNA971252". The corresponding BioProject number does not exist.

*Reply: We have released the BioProject PRJNA971252 linked with our study.*

6. Line 546-547. "All the code utilized in this study is available on GitHub at https://github.com/skinmicrobiome/ELSG". The webpage is unavailable.

*Reply: We have made the Github repository publicly available.*

7. Figure S1b: According to the manuscript, eukaryotic viral sequences were >1000bp (line 442). However, in the third boxplot of Figure S1(b), y-axis said the contig length is lower than 10-2 kb. Please address this discrepancy.

*Reply: We appreciate reviewer catching our mistake of the y-axis scale. We have fixed the axis scale in the revised manuscript and confirm that the shortest viral sequence included in our catalog is 1841bp, which is >1000bp*.

8. Figure 2: The figure has no scale of the bar graphs in the outermost layer.

*Reply: We have added the scale of the bar graphs in Figure 2b.*

9. In line 277, Fig. S5 has to be changed to Fig. S5a.

*Reply: We have changed accordingly.*

10. 441-443 "Eukaryotic viral sequences were detected by aligning the contigs from MEGAHIT to the nt database with BLASTn v2.8.032 by requiring >90% nucleotide identity, >1000 bp aligned sequence, and >70% total contig aligned". This sentence is redundant with the information provided in lines 432-434.

*Reply: We have deleted the redundancy in the revised manuscript.*

11. Line 522. What was minimum number of conspecific genomes for SNV analysis?

*Reply: The minimum number of conspecific genomes for SNV analysis was 10. We have included such information in the Methods section.*

Reviewer **#2:**

In this study, Shen and colleague collected skin samples from infants at age 2-3 months and 12 months for ultra-deep shotgun metagenomic sequencing to generate the early-life skin microbial genome catalog. Afterwards, based on the built catalogue, the author analyzed the property of the early-life skin microbiome. The comparisons between infants and mothers were conducted. Overall, this method is valid and the results of work are quite interesting and important for the future research from the early life.

Major comments:

1. From the title, the author aimed to build a catalogue of skin microbiome for infants. However, the infants are only from Australia, and the included subjects are not too many. As there have are other skin metagenomes from infants, I would suggest the author to search and include these public skin datasets from infants and incorporate them with the datasets newly sequenced in this study. Also, the rarefaction analysis showed that the number of species in the ELSG was not saturated, although the situation is better after removing the rare species.

*Reply: Most current studies describing ‘skin metagenomes’ used 16S rRNA amplicon sequencing (e.g., BioProject PRJNA833324). For the very few studies with shotgun metagenomic data, some contend with small sample sizes (e.g., n=2 within BioProject PRJNA573942, which primarily focuses on the gut microbiome, or n=10 from BioProject PRJNA937622 serving the purpose of protocol validation). Others focused on NICUs (neonatal intensive care units), as observed in PMID: 37422700, where the data deposition is still pending. We have scoured SRA/NCBI and ENA to find additional studies as that is our typical inclusive working style but were unable to find any comparable data. All of this increases the novelty of our findings, but we have also included the geographic site and single study as a limitation of our study in the revised manuscript.*

*Despite the single geographic site, our study still includes the largest number of subjects among all skin MAG studies. As the reviewer pointed out, our rarefaction analysis showed a saturated coverage of prevalent species on the infant skin and also some extent of individuality. Because individuality is the cause of our rarefaction curve not reaching saturation, adding more subjects would not solve the problem. Consistent with our findings, the existing gut microbiome catalogs (e.g., UHGG; PMID: 32690973), which combined multiple studies and contain >100,000 genomes, were also not able to reach saturation despite their larger sample sizes.*

2. The mother-to-infant transmission have been studied for the gut microbiome, as the author mentioned as well. Different from the gut microbiome that mainly comprises the strict anaerobic microbes, which means that these microbes are hard to survive in the environment, the skin microbiome are not anaerobic, and also fully exposed to the environment. Although the same species/strain shared by mothers and infants, how can the author exclude the possibility that these microbes are not transferred from environments to mothers and infants as they are shared the same environments. Therefore, the author should address in the discussion or be careful to use "transmission".

*Reply: We appreciate reviewer’s insights and have acknowledged the limitation of our study by addressing the possibility of other transmission sources besides mother in the Discussion section. We have also switched to the phrase “microbial sharing” when describing our conclusions. For example, in the revised manuscript, we discussed that our results “provided evidence for the mother-infant microbial sharing at the strain level and established the basis for future research on dissecting the transmission pathways of such sharing”.*

3. In "Comparison of taxonomic profiles between early-life and adult skin microbiome" part, the author should also describe and discuss the profile of microbiome from mother skin, and the differential taxa associated with mothers and infants. Only beta diversity comparison can provide quite limited information.

*Reply: We appreciate reviewer’s suggestion and have added description and discussion of the mother’s microbial profile in the revised manuscript:*

*“We also saw an increase in diversity from 2-3 months to 12 months at both body sites, with the richness of 12-month-old antecubital fossa resembling that of mothers.”*

*“While displaying the most similar profile to the 12-month-old antecubital fossa, the maternal antecubital fossa exhibited higher Actinobacteria and reduced Firmicutes (Fig. 3c)”*

*“When compared to maternal skin, 12-month-old infants showed significantly decreased Malassezia and increased Saccharomyces, aligning with the fungal diversity observed in the ELSG.”*

4. For microbial annotation, the author used the RefSeq database (release 99), which was released three years ago. I understand that that RefSeq is frequently updated, but I do not why the author used this very old release version, although I am not sure when the author conducted the analysis. The scientific reason has to be mentioned. However, I strongly recommend the author to use the updated release.

*Reply: We apologize for our mistake of falsely stating the RefSeq release number. In fact, the RefSeq database used in our study was first downloaded on May 11, 2022, and the latest release back then was release 211. We have corrected this accordingly in the revised manuscript.*

Minor comments:

Line 28: how does the "ultra-deep" shotgun sequences defined?

*Reply: The sequencing depth of our study is at least 3 times larger than any previous skin microbiome studies, so we used “ultra-deep” to highlight our deeper-than-ever shotgun sequencing. However, we recognize that an existing gut metagenomic study, which also used the term “ultra-deep” (PMID: 36604505), performed approximately 7 times deeper sequencing than our study. Thus, we have switched “ultra-deep” to “deep” in the revised manuscript to avoid confusion with gut microbiome studies.*

Line 263: only 7 species were shared by mother-infant?

*Reply: We performed SNV analysis only on species with MAGs assembled from at least 4 related infant-mother pairs for the benefit of statistical testing. To achieve more robust results, we restricted analyses to MAGs with >90% completeness and <5% contamination.*

Line 266: what the author means with "consistent with vertical transmission of skin microbes between mother and infant"? is it only based on the lower SNVs of paired mother-infants than unrelated ones? Is there a cut-off to define the transmission? Again, the "transmission" should be used with caution.

*Reply: We have revised this sentence to clarify that our finding indicates microbial sharing and not direct evidence of microbial transmission. We provide a fuller response to your Major Comment #2.*

Line 183: please update the release of RefSeq and see whether any difference rather than the 77% improvement.

*Reply: We have corrected the release number of the RefSeq database used in our study. See our response to your Major Comment #4, and thank you for catching our mistake.*

Line 491-495: is there any reason to exclude samples less than 800,000 classified reads and also 20% for meta diversity?

*Reply: A few samples yielded relatively few classified microbial reads because they often contain many human DNAs and low microbial biomass. Indeed, samples with less than 800,000 classified microbial reads have a median of 95% human reads compared with a median of 72% for the rest of the samples. A low microbial biomass could be due to several reasons, including bathing before sampling even though we did ask mothers not to bath their infants 24 hours before sample collection. To exclude the effects of unknown reasons resulting in low microbial biomass, we exclude samples with less than 800,000 classified reads in diversity calculation.*

*For beta diversity, we further removed taxa with less than 20% prevalence to focus on the taxa shared by at least multiple subjects so that the downstream analysis (e.g., PCoA, differential abundance) won’t be affected by individual-specific taxa.*

Line 411: is there any viral sequences that are removed after assessing by CheckV?

*Reply: No viral sequence was removed based on CheckV. Instead, CheckV quality statistics for all viral sequences were reported.*

Line 546: the link for code availability is not valid. Please update.

*Reply: The link for codes has been fixed.*

**Second round of review**

**Reviewer 1**

All my questions were thoroughly addressed in the revised version of manuscript. Now I recommend this manuscript for publication.

**Reviewer 2**

The authors have addressed all my concerns based on the original submission.
